# Recombinant LH supplementation improves cumulative live birth rates in the GnRH antagonist protocol: a multicenter retrospective study using a propensity score-matching analysis

**DOI:** 10.1186/s12958-022-00985-4

**Published:** 2022-08-08

**Authors:** Meng Wang, Rui Huang, Xiaoyan Liang, Yundong Mao, Wenhao Shi, Qian Li

**Affiliations:** 1 Reproductive Medicine Center, The Sixth Affiliated Hospital, Sun Yat-Sen University, Guangzhou, 510000 China; 2 Reproductive Medicine Center, State Key Laboratory of Reproductive Medicine, Center of Clinical Reproductive Medicine, First Affiliated Hospital of Nanjing Medical University, Nanjing, 210000 China; 3 Reproductive Medicine Center, Northwest Women’s and Children’s Hospital, Xi’an 710000, China

**Keywords:** r-LH supplementation, GnRH antagonist protocol, Cumulative live birth rate, Live birth rate, Embryo development, Controlled ovarian stimulation

## Abstract

**Background:**

Luteinizing hormone (LH) is critical in follicle growth and oocyte maturation. However, the value of recombinant LH (r-LH) supplementation to recombinant follicle stimulating hormone (r-FSH) during controlled ovarian stimulation in the gonadotrophin releasing hormone (GnRH) antagonist regimen is controversial.

**Methods:**

This multicenter retrospective cohort study recruited 899 GnRH antagonist cycles stimulated with r-LH and r-FSH in 3 reproductive centers and matched them to 2652 r-FSH stimulating cycles using propensity score matching (PSM) for potential confounders in a 1:3 ratio. The primary outcome was the cumulative live birth rate (CLBR) per complete cycle.

**Results:**

The baseline characteristics were comparable in the r-FSH/r-LH and r-FSH groups after PSM. The r-FSH/r-LH group achieved a higher CLBR than the r-FSH group (66.95% vs. 61.16%, *p* = 0.006). R-LH supplementation also resulted in a higher 2-pronuclear embryo rate, usable embryo rate, and live birth rate in both fresh embryo transfer cycles and frozen-thawed embryo transfer (FET) cycles. No significant differences were found in the rate of moderate and severe ovarian hyperstimulation syndrome (OHSS), or cycle cancellation rate in the prevention of OHSS.

**Conclusions:**

R-LH supplementation to r-FSH in the GnRH antagonist protocol was significantly associated with a higher CLBR and live birth rate in fresh and FET cycles, and improved embryo quality without increasing the OHSS rate and cycle cancellation rate.

**Supplementary Information:**

The online version contains supplementary material available at 10.1186/s12958-022-00985-4.

## Background

Controlled ovarian stimulation (COS) is the first and critical procedure of in vitro fertilization (IVF) or intracytoplasmic sperm injection (ICSI). Gonadotropin releasing hormone (GnRH) antagonists are applied to conveniently downregulate the pituitary gland to reduce endogenous gonadotropins without the flare-up effect. Thus, follicle development is under the control of exogenous gonadotropins.

According to the “two cell-two gonadotropins” theory, follicle stimulating hormone (FSH) synergizes with luteinizing hormone (LH) to promote folliculogenesis and oocyte maturation. FSH stimulates granulosa cells to produce estradiol from androgen transformed from cholesterol by theca cells in response to LH stimulation. Recombinant FSH (r-FSH) alone can induce follicular development after pituitary downregulation, probably because residual endogenous LH secretion is not completely suppressed, thereby participating in the follicular development. However, follicular development was undermined [1], decidualization of the endometrium was affected, and implantation of embryos was disturbed [2] in the absence of exogenous LH. Therefore, the addition of exogenous recombinant LH (r-LH) in combination with FSH was inferred to promote the normal development of follicles. While conflicting opinions regarding this effect have been published.

A real-world study involving 9787 cycles suggested that LH supplementation for moderate and severe poor ovarian responders can improve the cumulative live birth rate (CLBR) [3]. When comparing the 2^nd^ ICSI cycles stimulated by r-FSH and r-LH in the GnRH antagonist protocol with the 1^st^ cycles stimulated with r-FSH for 228 cycles, the implantation rate was increased by 8% in the 2^nd^ cycles (1^st^ cycle: 18.57 ± 0.52 vs. 2^nd^ cycle: 26.47 ± 0.62, *p* < 0.001) [4]. A study involving 320 cycles receiving the GnRH antagonist treatment [5] revealed that exogenous LH supplementation resulted in more oocytes retrieved (5.60 vs. 3.97, *p* = 0.04) and good-quality embryos (3.07 vs. 1.93, *p* = 0.01) for patients over 40 years old compared with the r-FSH alone group. Another study involving 1565 cycles [6] concluded that r-LH supplementation based on the r-FSH stimulation was associated with increased pregnancy rate (61% vs. 54%, *p* = 0.006), live birth rate (49% vs. 42%, *p* = 0.01), fertilization rate (74% vs. 72%, *p* = 0.04), and implantation rate (41% vs. 37%, *p* = 0.03). A meta-analysis [7] concluded that r-LH combined with r-FSH probably improved ongoing pregnancy rates (OR 1.20, 95% CI 1.01 to 1.42) compared to using r-FSH alone.

However, a systematic review [8] has suggested that ovarian stimulation with r-LH and r-FSH did not differed from stimulation with r-FSH in terms of the number of oocytes retrieved, implantation rate and clinical pregnancy rate in women undergoing the GnRH antagonist cycles. A meta-analysis [9] showed that no significant difference presented in ongoing pregnancy rate, pregnancy rate, OHSS rate between LH supplementation and r-FSH alone in women.

The abovementioned studies did not reach a consensus on the value of LH supplementation. Furthermore, its effects on embryo development and its impact on the CLBR are less systematically evaluated. Thus, the present study aimed to investigate whether patients undergoing the GnRH antagonist protocol could benefit from r-LH supplementation in terms of embryo development, live birth rates in fresh cycles and frozen-thawed embryo transfer (FET) cycles, as well as the CLBR in complete cycles (when at least a live birth was achieved or all embryos from the same retrieval cycle were transferred) in 3 reproductive centers.

## Methods

### Study population and design

This multicenter retrospective cohort study recruited the autologous IVF/ICSI cycles using GnRH antagonist protocol conducted at the Reproductive Medicine Center of the Sixth Affiliated Hospital of Sun Yat-sen University, Northwest Women’s and Children’s Hospital, and Jiangsu Provincial Hospital from January 2014 to December 2018. The study group underwent ovarian stimulation with r-FSH and r-LH. R-LH was added on the day of GnRH-antagonist had been started. The control group received ovarian stimulation with r-FSH.

Only the first oocyte retrieval cycles of a patient and the subsequent FET cycles were included. Cycles were excluded if (1) the female partner underwent recurrent spontaneous miscarriages or suffered from hydrosalpinx, intrauterine adhesions, uterine malformations, submucosal myoma, adenomyosis or thyroid dysfunction; (2) either of the spouses presented chromosomal abnormalities; (3) human menopausal gonadotropin, letrozole or clomiphene citrate were used for ovarian stimulation; (4) cycles included in vitro matured oocytes, frozen-thawed embryos, biopsied embryos. Oocyte retrieval, fresh embryo transfer, FET and complete cycles (at least a live birth was achieved or all embryos from the same retrieval cycle were transferred) were included in the analyses. Cycles were followed up until December 2020. Subgroup analysis were conducted for the normal responders who were younger than 40 years old and the number of oocytes retrieved were between 4 and 15 [10].

This project was approved by Ethics Committees at the Sixth Affiliated Hospital of Sun Yat-sen University (2020ZSLYEC-295), Northwest Women’s and Children’s Hospital (2,019,013), and Jiangsu Provincial Hospital (2020-SR-046). The written informed consents were waived due to the retrospective nature of this study.

### Controlled ovarian stimulation procedures and embryo evaluation

Fixed GnRH antagonist protocol (GnRH antagonist started on day 5 of r-FSH stimulation) and flexible GnRH antagonist protocol (GnRH antagonist started when the mean diameter of dominant follicles reached 12 mm) [11] were adopted for controlled ovarian stimulation following the routine of the three reproductive centers. R-FSH of 100 to 300 IU/day was administered according to the individual characteristics, such as anti-müllerian hormone, basal FSH, LH, estradiol, progesterone, and antral follicle counts (AFC) on days 2–3 of the menstrual cycle. Whether r-LH was supplemented was determined by the specialties.

Follicular growth was evaluated by serum concentration of estradiol, progesterone, FSH, and LH, and transvaginal ultrasonography. Once the diameters of three dominant follicles ≥ 17 mm or the diameters of two dominant follicles ≥ 18 mm, human chorionic gonadotrophin was injected, and oocytes were retrieved after 36-38 h. Most fresh embryos were transferred on day 3. Freezing all embryos was adopted when there was a risk of ovarian hyperstimulation syndrome (OHSS) [12], thin endometrium and elevated progesterone. Cleavaged embryos were evaluated by Scott’s criteria [13]: grades I and II with ≥ 4 cells were usable; with ≥ 6 cells were of good quality. Blastocysts (days 5 and 6) were evaluated by Gardner’s system [14]: blastocysts graded as 3–6, and inner cell mass and trophectoderm assessed as AA, AB, AC, BA, BB, BC, CA, or CB were usable embryos, grade 3–6 blastocysts graded with AA, AB, BA, and BB were good-quality embryos.

Luteal phase support was performed with oral or vaginal progesterone until 8 weeks of gestation.

### Outcome measures

The primary outcome was the CLBR which referred to the probability of achieving at least 1 live birth in a complete cycle.

Secondary outcomes assessed included number of oocytes retrieved, number of two-pronuclear (2PN) embryos and 2PN embryo rate after IVF, number of 2PN embryos and 2PN embryo rate after ICSI, usable embryo number and rate, good-quality embryo number and rate, mild/moderate OHSS [12] rate and cycle cancellation rate in prevention of OHSS. In terms of pregnancy outcomes, clinical pregnancy rate and live birth rate were evaluated. Clinical pregnancy was defined as the observation of gestational sac(s) through a transvaginal probe. Live birth was defined as a live-born infant(s) after 28 gestational weeks.

### Statistical analysis

Propensity score-matching analysis (PSM) and data analysis were conducted with SAS version 9.4 (SAS Institute, Cary, NC, USA). Patients of the study group and the control group were randomly matched with the 1:3 nearest neighbor matching method. The covariates included maternal age, paternal age, maternal body mass index (BMI), infertility factors, infertility type, basal FSH, AFC, and fertilization type.

Continuous variables that followed normal distribution were expressed as mean values (± standard deviations, SDs), and compared by student’s t-test. Data with skewed distribution were described as medians (quartiles) and Mann–Whitney U test was adopted for comparisons. Categorical variables were described as counts (percentages) and compared by Pearson Chi-square test. *P* < 0.05 was regarded as statistically significant.

## Results

**Fig. 1:**
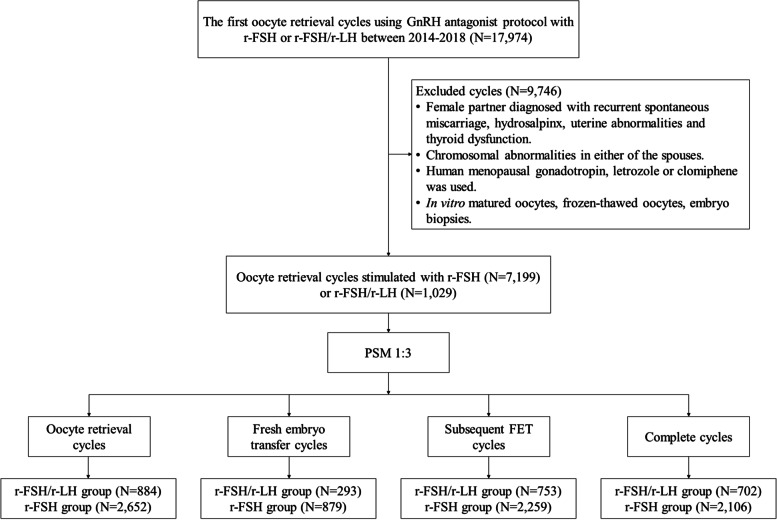
The flowchart of the study

The process of inclusion and exclusion is shown in Fig. 1. A total of 8,228 oocyte retrieval cycles met the inclusion criteria, of which 1,029 cycles received r-FSH/r-LH administration. After PSM, 884 oocyte retrieval cycles that received r-FSH/r-LH treatment were matched with 2,652 r-FSH stimulated cycles, 293 fresh embryo transfer cycles with r-FSH/r-LH treatment were matched with 879 r-FSH stimulated cycles, 753 FET cycles with r-FSH/r-LH treatment were matched with 2,259 r-FSH stimulated cycles, and 702 complete cycles with r-FSH/r-LH treatment were matched with 2,106 r-FSH stimulated cycles.

### Baseline characteristics

The baseline characteristics of the oocyte retrieval cycles are presented in Table 1. Parental ages, female BMI, infertility factors, infertility type, AFC, basal LH and fertilization type were not statistically significant after PSM. The basal FSH of the study group was close to that of the control group (6.48 [5.48, 7.60] vs. 6.26 [5.32, 7.52]), although the difference was statistically significant (*p* = 0.012). Table 2 shows the baseline characteristics of the fresh embryo transfer cycles, Table 3 shows those of the FET cycles, and Table 4 displays those of the complete cycles. The distributions of these characteristics were comparable between the two groups after PSM except that the basal LH levels were significantly lower in the study group in Table 3 (*p* = 0.015) and Table 4 (*p* = 0.006). On the day of triggering, the estradiol, LH, progesterone level, and number of developed follicles were significantly higher in the study group from Tables 1, 2, 3, 4.

**Table 1:** Characteristics of oocyte retrieval cycles

Variables	r-FSH/r-LH	r-FSH	*p*
No. of cycles	884	2652	
Female age (year)	30.68 ± 5.24	30.70 ± 4.58	0.922
Male age (year)	32.62 ± 6.24	32.64 ± 5.32	0.928
Female BMI (kg/m^2^)	22.57 ± 3.03	22.62 ± 3.38	0.717
Infertility factor, n (%)			0.817
Ovulatory disorder	227 (25.68%)	705 (26.58%)	
Diminished ovary reserve	99 (11.20%)	323 (12.18%)	
Pelvic and tubal disease	336 (38.01%)	987 (37.22%)	
Endometriosis	65 (7.35%)	173 (6.52%)	
Male factor	157 (17.76%)	464 (17.50%)	
Infertility type, n (%)			0.768
Primary infertility	506 (57.24%)	1533 (57.81%)	
Secondary infertility	378 (42.76%)	1119 (42.19%)	
Basal FSH (IU/L)	6.48 (5.48, 7.60)	6.26 (5.32, 7.52)	0.012
Basal LH (IU/L)	4.87 (3.50,6.69)	5.01 (3.50,7.22)	0.121
Basal AFC	18.00 (11.50, 20.00)	16.00 (9.00, 23.00)	0.143
Fertilization type, n (%)			0.984
IVF	586 (66.29%)	1759 (66.33%)	
ICSI	298 (33.71%)	893 (33.67%)	
E2 on trigger Day (pg/ml)	3475.50 (2113.00,5012.00)	2973.00 (1815.00,5062.00)	0.002
LH on trigger Day (IU/L)	2.67 (1.75,3.93)	2.40 (1.35,3.95)	< 0.001
P on trigger Day (ng/ml)	1.95 (0.80,3.76)	1.01 (0.64,1.62)	< 0.001
Follicle counts on trigger Day	15.00 (11.00,20.00)	14.00 (8.00,19.00)	< 0.001

Data are displayed as mean ± standard deviation and median (interquartile range) for continuous variables and n (%) for categorical variables

*BMI* body mass index, r-*FSH* recombinant follicle-stimulating hormone, *r-LH* recombinant luteinizing hormone, *AFC* antral follicle count, *IVF* in-vitro fertilization, *ICSI* intracytoplasmic sperm injection, *E2* estradiol, *P* progestin

**Table 2:** Characteristics of the fresh embryo transfer cycles

Variables	r-FSH/r-LH	r-FSH	*p*
No. of cycles	293	879	
Female age (year)	30.91 ± 5.19	30.98 ± 4.53	0.838
Male age (year)	32.69 ± 6.15	32.65 ± 5.11	0.907
Female BMI (kg/m^2^)	22.86 ± 3.31	22.85 ± 3.47	0.947
Infertility factor, n (%)			0.813
Ovulatory disorder	74 (25.26%)	221 (25.14%)	
Diminished ovary reserve	38 (12.97%)	136 (15.47%)	
Pelvic and tubal disease	106 (36.18%)	292 (33.22%)	
Endometriosis	16 (5.46%)	52 (5.92%)	
Male factor	59 (20.14%)	178 (20.25%)	
Infertility type, n (%)			0.973
Primary infertility	173 (59.04%)	520 (59.16%)	
Secondary infertility	120 (40.96%)	359 (40.84%)	
Basal FSH (IU/L)	6.71 (5.74, 7.77)	6.53 (5.58, 7.88)	0.448
Basal LH (IU/L)	4.93 (3.48, 6.50)	4.93 (3.36, 6.92)	0.696
AFC	16.00 (10.00, 20.00)	14.00 (8.00, 22.00)	0.259
Fertilization type, n (%)			0.972
IVF	193 (65.87%)	580 (65.98%)	
ICSI	100 (34.13%)	299 (34.02%)	
E2 on trigger Day (pg/ml)	2438.50 (1654.00, 3297.00)	2151.00 (1467.00, 3119.00)	0.016
LH on trigger Day (IU/L)	2.95 (1.91, 4.56)	2.34 (1.36, 3.95)	< 0.001
P on trigger Day (ng/ml)	1.20 (0.63, 2.96)	0.76 (0.50, 1.12)	< 0.001
Follicle counts on trigger Day	12.00 (9.00, 15.00)	11.00 (7.00, 15.00)	0.016

Data are displayed as mean ± standard deviation and median (interquartile range) for continuous variables and n (%) for categorical variables

*BMI* body mass index, r-*FSH* recombinant follicle-stimulating hormone, *r-LH* recombinant luteinizing hormone, *AFC* antral follicle count, *IVF* in-vitro fertilization, *ICSI* intracytoplasmic sperm injection, *E2* estradiol, *P* progestin

**Table 3:** Characteristics of the FET cycles

Variables	r-FSH/r-LH	r-FSH	*p*
No. of cycles	753	2259	
Female age (year)	30.18 ± 4.81	30.23 ± 4.33	0.798
Male age (year)	32.22 ± 5.99	32.32 ± 5.04	0.675
Female BMI (kg/m^2^)	22.37 ± 2.89	22.37 ± 3.20	0.991
Infertility factor, n (%)			0.977
Ovulatory disorder	211 (28.02%)	641 (28.38%)	
Diminished ovary reserve	50 (6.64%)	164 (7.26%)	
Pelvic and tubal disease	309 (41.04%)	905 (40.06%)	
Endometriosis	51 (6.77%)	154 (6.82%)	
Male factor	132 (17.53%)	395 (17.49%)	
Infertility type, n (%)			0.983
Primary infertility	433 (57.50%)	1300 (57.55%)	
Secondary infertility	320 (42.50%)	959 (42.45%)	
Basal FSH (IU/L)	6.31 (5.39,7.47)	6.22 (5.25,7.42)	0.178
Basal LH (IU/L)	4.98 (3.58, 6.78)	5.23 (3.64, 7.52)	0.015
AFC	19.00 (14.00,21.00)	18.00 (10.00,24.00)	0.665
Fertilization type, n (%)			0.786
IVF	517 (68.66%)	1539 (68.13%)	
ICSI	236 (31.34%)	720 (31.87%)	
E2 on trigger Day (pg/ml)	4427.00 (2789.00, 5439.00)	3833.00 (2341.00, 5983.00)	0.012
LH on trigger Day (IU/L)	2.38 (1.52, 3.67)	2.25 (1.27, 3.81)	0.009
P on trigger Day (ng/ml)	2.58 (1.00, 4.19)	1.18 (0.77, 1.88)	< 0.001
Follicle counts on trigger Day	17.00 (13.00, 23.00)	15.00 (10.00, 21.00)	< 0.001

Data are displayed as mean ± standard deviation and median (interquartile range) for continuous variables and n (%) for categorical variables

*FET* frozen-thawed embryo transfer, *BMI* body mass index, r-*FSH* recombinant follicle-stimulating hormone, *r-LH* recombinant luteinizing hormone, *AFC* antral follicle count, *IVF* in-vitro fertilization, *ICSI* intracytoplasmic sperm injection, *E2* estradiol, *P* progestin

**Table 4:** Characteristics of the complete cycles

Variables	r-FSH/r-LH	r-FSH	*p*
No. of cycles	702	2106	
Female age (year)	30.56 ± 5.22	30.55 ± 4.65	0.940
Male age (year)	32.43 ± 6.12	32.37 ± 5.17	0.826
Female BMI (kg/m^2^)	22.55 ± 3.00	22.55 ± 3.28	0.989
Infertility factor, n (%)			0.946
Ovulatory disorder	184 (26.21%)	561 (26.64%)	
Diminished ovary reserve	80 (11.40%)	258 (12.25%)	
Pelvic and tubal disease	267 (38.03%)	798 (37.89%)	
Endometriosis	50 (7.12%)	137 (6.51%)	
Male factor	121 (17.24%)	352 (16.71%)	
Infertility type, n (%)			0.895
Primary infertility	405 (57.69%)	1221 (57.98%)	
Secondary infertility	297 (42.31%)	885 (42.02%)	
Basal FSH (IU/L)	6.54 (5.50,7.69)	6.36 (5.40,7.65)	0.187
Basal LH (IU/L)	4.92 (3.50, 6.79)	5.20 (3.68, 7.44)	0.006
AFC	18.00 (12.00,20.00)	17.00 (9.00,24.00)	0.514
Fertilization type, n (%)			0.612
IVF	461 (65.67%)	1405 (66.71%)	
ICSI	241 (34.33%)	701 (33.29%)	
E2 on trigger Day (pg/ml)	3613.00 (2069.50, 5111.00)	3084.00 (1832.00, 5336.00)	0.005
LH on trigger Day (IU/L)	2.60 (1.68, 3.84)	2.41 (1.40, 4.14)	0.021
P on trigger Day (ng/ml)	1.93 (0.80, 3.88)	1.03 (0.66, 1.73)	< 0.001
Follicle counts on trigger Day	16.00 (11.00, 21.00)	14.00 (9.00, 20.00)	< 0.001

Data are displayed as mean ± standard deviation and median (interquartile range) for continuous variables and n (%) for categorical variables

*BMI* body mass index, r-*FSH* recombinant follicle-stimulating hormone, *r-LH* recombinant luteinizing hormone, *AFC* antral follicle count, *IVF* in-vitro fertilization, *ICSI* intracytoplasmic sperm injection, *E2* estradiol, *P* progestin

### Embryo development

The comparisons of embryo development between the study and control groups are presented in Table 5. The study group had fewer retrieved oocytes than the control group (*p* < 0.001). The number of 2PN embryos was comparable between oocytes fertilized by IVF and those fertilized by ICSI. Thus, the study group presented a statistically higher 2PN embryo rate than the control group (IVF 2PN rate: *p* < 0.001; ICSI 2PN rate: *p* = 0.003) (Table 5). The number of usable embryos and good-quality embryos were comparable between the two groups. While the usable embryo rate in 2PN embryos was significantly higher in the study group (*p* < 0.001), no significant difference existed between the good-quality embryo rates of the two groups. Furthermore, the rate of mild or moderate OHSS was similar between the groups (*p* = 0.864). The cycle cancellation rate in the prevention of OHSS was not significantly different between the two groups.

**Table 5:** Number of oocytes retrieved and embryo assessment

Variables	r-FSH/r-LH	r-FSH	*p*
No. of cycles	884	2652	
Oocyte retrieval	10.00 (7.00, 14.00)	12.00 (7.00, 17.00)	< 0.001
IVF 2PN number	7.00 (4.00, 10.00)	7.00 (4.00, 11.00)	0.572
IVF 2PN rate (%)	90.00% (77.78%, 100.00%)	86.96% (75.00%, 100.00%)	< 0.001
ICSI 2PN number	6.00 (4.00, 10.00)	7.00 (4.00, 10.00)	0.251
ICSI 2PN rate (%)	81.82% (62.50%, 93.75%)	75.00% (60.00%, 88.89%)	0.003
Usable embryos	6.00 (3.00, 9.00)	6.00 (3.00, 9.00)	0.887
Usable embryo rate (%)	92.31% (75.00%, 100.00%)	87.50% (66.67%, 100.00%)	< 0.001
Good-quality embryo	4.00 (2.00, 7.00)	4.00 (2.00, 7.00)	0.320
Good-quality embryo rate (%)	70.59% (50.00%, 90.00%)	69.23% (50.00%, 87.50%)	0.427
Mild/moderate OHSS rate (%)	3.05% (27/884)	2.94% (78/2652)	0.864
Cycle cancellation rate due to OHSS (%)	30.88% (273/884)	27.53% (730/2652)	0.055

Data are displayed as median (interquartile range) for continuous variables because they follow the skewed distribution and % (n) for categorical variables

*r-FSH* recombinant follicle-stimulating hormone, *r-LH* recombinant luteinizing hormone, *IVF* in-vitro fertilization, *2PN* 2 pronuclear, *ICIS* intracytoplasmic sperm injection, *OHSS* ovarian hyper-stimulation syndrome

### Pregnancy outcomes

After fresh embryo transfer, 50.85% cycles of the study group, and 49.49% cycles of the control group achieved clinical pregnancy (*p* = 0.686). The live birth rate in the fresh cycles was significantly higher in the study group than in the control group (39.93% vs. 28.10%, *p* < 0.001).

For FET cycles, the clinical pregnancy rate in the study group was 63.75% versus that of 61.97% in the control group, and no significant difference was observed. The study group obtained a significantly higher live birth rate than the control group (51.53% vs. 43.16%, *p* < 0.001).

The CLBR in complete cycles of the study group was also significantly higher than that of the control group (66.95% vs. 61.16%, *p* = 0.006).

### Subgroup analysis of normal responders

Subgroup analysis of normal responders were conducted. Among the normal responders, 616 oocyte retrieval cycles of the study group were matched with 1,848 cycles of the control group. No significantly differences existed between the baseline characteristics of the study groups and the control groups of the oocyte retrieval cycles (Supplementary table 1), fresh embryo transfer cycles (Supplementary table 2), FET cycles (Supplementary table 3), and complete cycles (Supplementary table 4) except that the basal LH in the study group were significantly higher than that in the control group of the FET cycles (*p* = 0.041, Supplementary table 3). While the E2, LH, P and the number of developed follicles on the day of triggering were significantly higher in the study groups (Supplementary table 1, 2, 3, 4).

Consistent with the outcomes of the whole group analysis, the study groups of the normal responders achieved significantly better prognosis than the control groups. The number of oocytes retrieved were close in the two groups. The number of 2PN embryos fertilized by IVF (*p* = 0.003) and the rate of 2PN embryos by IVF (*p* < 0.001) were higher in the study group of normal responders (Supplementary Table 5). The number of oocytes retrieved, number and rate of 2PN embryos fertilized by ICSI, number and rate of usable embryos and good-quality embryos, and mild/moderate OHSS rate were comparable between the two groups. However, the cycle cancellation rate in the prevention of the study group was significantly higher in the study group (*p* < 0.001) (Supplementary table 5).

The clinical pregnancy rates in the fresh embryo transfer cycles were comparable (*p* = 0.413) between the study group (55.79%) and the control group (52.75%). However, the live birth rate in the fresh cycles was significantly higher in the study group than in the control group (42.98% vs.33.06%, *p* = 0.005).

No significantly difference was found in the clinical pregnancy rate in the FET cycles between the two groups (study group: 65.88% vs. control group: 63.27%, *p* = 0.287), But the study group in the FET cycles obtained a significantly higher live birth rate than the control group (53.73% vs. 45.56%, *p* = 0.001).

The study group also achieved significantly higher CLBR than that of the control group (69.79% vs. 61.56%, *p* = 0.001).

## Discussion

Numerous publications [3, 7, 8, 15–20] have proven that r-FSH alone is capable of inducing satisfactory follicle development during controlled ovarian stimulation. Although LH is critical for follicle growth and oocyte maturation, the benefit of r-LH supplementation in the GnRH antagonist regimen remains disputable. In this multicenter retrospective cohort study, we investigated the effects of r-LH supplementation on the whole process of IVF/ICSI in the same cohort for the first time. R-LH supplementation was found to be associated with improved embryo development, live birth rates in both fresh and FET cycles, and the CLBR in complete cycles in patients receiving the GnRH antagonist regimen. The occurrence rates of OHSS and cycle cancellation in the prevention of OHSS were not increased.

The effects of r-LH supplementation on embryo development, OHSS rate, and cycle cancellation rate have not been clearly investigated. Although a randomized study reported a lower OHSS incidence and lower cycle cancellation rate in the r-LH supplementation group that was downregulated by GnRH agonists [21], no significant differences were observed in the present study. The different conclusions might be attributed to the GnRH antagonist protocol in our study, which reduced the occurrence of OHSS compared with the GnRH agonist protocol [22]. However, the subgroup analysis of normal responders revealed a higher cycle cancellation rate in the study group. It was probably because that the E2 level on the trigger day was significantly higher in the study group, thus the embryo transfer was more frequently cancelled in the study group to prevent OHSS.

A prospective randomized study, which focused on cycles in which the GnRH antagonist was administered, reported that the number of oocytes retrieved was similar whether r-LH was supplemented or not [19]. However, another prospective randomized study [23] showed that the number of oocytes recovered was relatively lower in the r-FSH/r-LH group (5.33 ± 4.8 vs. 7.00 ± 3, *p* > 0.05), whose trend was consistent with the results of our study. This phenomenon suggested that r-LH supplementation did not help to improve ovarian response.

Our data showed that r-LH supplementation was associated with an increased normal fertilization rate (2-pronuclear embryo rate of both IVF and ICSI), usable embryo rate, and live birth rate in FET cycles. These finding were consistent with the conclusions of Paterson’s [6] and Lisi’s study [24]. In the previous studies, LH was proven to promote folliculogenesis by (i) facilitating the synthesis of androgens for the production of estradiol and the induction of FSH receptor expression in granulosa cells [25]; (ii) recruiting local growth factors, such as EGF, GDF9, and TGF-β to promote oocyte maturation [23, 26]; (iii) decreasing the cumulus apoptosis rate [23]; (iv) resuming meiosis and ovulation [15, 27]. Thus, r-LH supplementation contributed to improve the quality of oocytes and promote embryogenesis.

Furthermore, the present study showed that the live birth rate in the fresh cycles was elevated in the study group, which was in accordance with previous studies showing that a higher live birth rate was achieved when r-LH was supplemented in the GnRH antagonist protocol [4–7]. Exposure to low endogenous LH by downregulation leads to stagnation of endometrial growth [28], decreasing endometrium receptivity [2], and decreasing the implantation rate [28]. The disturbance can be rescued by LH receptor stimulation through mid-cycle HCG supplementation [28]. LH supplementation probably achieved the same effects on endometrium as the HCG supplementation.

Many researches have discussed the “LH activity” supplementation on progesterone levels on the triggering day, but no consensus has been reached. In five previous studies [16, 19, 29–31], no significant difference in serum progesterone was observed between groups with or without r-LH supplementation during COS. One study [32] suggested the progesterone level was significantly lower in r-LH supplied group, and the number of follicles was significantly reduced. Three other studies [33–35] found that when LH activity (hCG) was provided during the late follicular phase, serum P-values were significantly increased. We also observed that the progesterone levels were significantly higher in the study group of the present study, which might result from the elevated number of developed follicles after r-LH supplementation. The inconsistency suggests that the effect of r-LH supplementation on progesterone may vary among patients with different characteristics [36], which requires further analysis in a larger sample size of patients.

The clinical pregnancy rates in fresh and FET cycles were comparable between the two groups, indicating a higher miscarriage rate in the r-FSH group, which might suggest the unsatisfactory developmental potential of embryos.

Traditionally, live birth rate per embryo transfer has been reported as the success rates of IVF/ICSI [37]. However, embryo freezing and thawing becomes increasingly universal that live birth rate of a single embryo transfer cycle is not adequate to evaluate the effect of the IVF/ICSI treatment. CLBR describes the probability that a person will deliver at least one baby after transferring all fresh and frozen embryos from the same oocyte retrieval cycle [38]. Not only was the quality of embryos assessed, but the number of usable embryos was also evaluated. Therefore, the CLBR has been regarded as the most valuable patient-centered outcome to assess the success of IVF/ICSI treatment [39]. However, the impact of r-LH supplementation on the CLBR has been less investigated perhaps because the calculation of the CLBR is more complicated than the live birth rate in an embryo transfer cycle. The former requires the selection of complete cycles (all embryos form an oocyte retrieval cycle were used up or at least a live birth was achieved) and also the integration of results of a series of fresh and frozen embryo transfer cycles. There is only one real-world study focusing on poor ovarian responders and reporting that the CLBR of moderate and severe poor ovarian responders is improved when r-LH is provided [3]. Our study is the first to report the effect of r-LH supplementation on CLBR in patients receiving the GnRH antagonist protocol. The conclusion suggests that the CLBR is elevated in r-FSH/r-LH stimulated patients with GnRH antagonist pituitary downregulation, perhaps through elevated oocyte quality, promoted embryo developmental potential, and optimized decidualization and receptivity.

Based on the consensus on LH supplementation among the Asia Pacific Fertility Advisory Group in 2011 [40], LH supplementation has been recommended to patients with central ovarian failure, poor ovarian response histories with < 4 oocytes with FSH levels ≥ 300 IU/day, and unsatisfactory responses to the current COS cycle. Furthermore, patients aged > 35 years should consider r-LH supplementation due to the potential of poor or suboptimal responses, and the decreased bioactivity of endogenous LH [40]. Our study, on the other hand, provides new evidence on the value of r-LH supplementation for common patients receiving COS through the GnRH antagonist protocol.

Considered to the affection of the intrinsic nature of retrospective research, especially selection bias of LH supplementation, we adopted PSM to balance the differences between the two groups, including maternal age, paternal age, maternal BMI, infertility factors, infertility type, basal FSH, AFC, and fertilization type. After PSM, the bias was reduced as much as possible, so that the data between the two groups were comparable and the research results were reliable to a certain extent.

The first strength of this study is that the CLBR was set as the primary outcome, thereby providing a global overview of the effects of r-LH supplementation in such individuals. Secondly, the present study provides a robust analysis on the suitable populations for r-LH supplementation based on data from reproductive centers in three tertiary hospitals. The r-LH supplementation has been conventionally recommended to patients with central ovarian failure, poor ovarian response histories, and unsatisfactory responses to the current COS, or patients aged > 35 years [33]. But our study extended the suitable populations to the patients undergoing the GnRH antagonist protocol. No matter the individuals were normal responders or not, it is helpful to improve the prognosis of these people.

## Conclusions

In conclusion, r-LH supplementation to r-FSH in the GnRH antagonist protocol resulted in a statistically significantly higher CLBR, live birth rate in fresh and FET cycles, and better embryos without increasing the OHSS rate and cycle cancellation rate. The effects might have been achieved through higher quality embryos, the promotion of embryo developmental potential, and the optimization of decidualization and receptivity.

## Supplementary Information

**Additional file 1: Supplementary table 1.** Characteristics of oocyte retrieval cycles of normal responders.** Supplementary table 2.** Characteristics of the fresh embryo transfer cycles of normal responders. **Supplementary table 3.** Characteristics of the FET cycles of normal responders. **Supplementary table 4.** Characteristics of the complete cycles of normal responders. **Supplementary table 5.** Number of oocytes retrieved and embryo assessment.

## Data Availability

The datasets used and/or analyzed during the current study are available from the corresponding author on reasonable request.

## Abbreviations

r-LH: Recombinant Luteinization hormone
GnRH: Gonadotrophin releasing hormone
r-FSH: Recombinant-follicle stimulation hormone
PSM: Propensity score matching
CLBR: Cumulative live birth rate
FET: Frozen-thawed embryo transfer
OHSS: Ovarian hyperstimulation syndrome
COS: Controlled ovarian stimulation
IVF: In vitro fertilization
ICSI: Intracytoplasmic sperm injection
AFC: Antral follicle counts
2PN: 2 Pronuclear
BMI: Body mass index
SD: Standard deviation
