# Supplementary material for: Recombinant LH supplementation improves cumulative live birth rates in the GnRH antagonist protocol: a multicenter retrospective study using a propensity score-matching analysis
Source: Reprod Biol Endocrinol. 2022 Aug 8;20:114. doi: 10.1186/s12958-022-00985-4 (PMC9358814; doi:10.1186/s12958-022-00985-4)
Supplement: Supplementary file 1 — Additional file 1: Supplementary table 1. Characteristics of oocyte retrieval cycles of normal responders. Supplementary table 2. Characteristics of the fresh embryo transfer cycles of normal responders. Supplementary table 3. Characteristics of the FET cycles of normal responders. Supplementary table 4. Characteristics of the complete cycles of normal responders. Supplementary table 5. Number of oocytes retrieved and embryo assessment. [file 12958_2022_985_MOESM1_ESM.docx]

**Supplementary table 1** Characteristics of oocyte retrieval cycles of normal responders

| **Variables** | **r-FSH/r-LH** | **r-FSH** | ***p*** |
| --- | --- | --- | --- |
| No. of cycles | 616 | 1848 |  |
| Female age (year) | 29.79 ± 4.15 | 29.76 ± 3.74 | 0.861 |
| Male age (year) | 31.63 ± 5.42 | 31.61 ± 4.48 | 0.954 |
| Female BMI (kg/m^2^) | 22.48 ± 3.09 | 22.50 ± 3.30 | 0.876 |
| Infertility factor, n (%) |  |  | 0.741 |
| Ovulatory disorder | 162 (26.30%) | 504 (27.27%) |  |
| Diminished ovary reserve | 41 (6.66%) | 149 (8.06%) |  |
| Pelvic and tubal disease | 246 (39.94%) | 717 (38.80%) |  |
| Endometriosis | 53 (8.60%) | 143 (7.74%) |  |
| Male factor | 114 (18.51%) | 335 (18.13%) |  |
| Infertility type, n (%) |  |  | 0.633 |
| Primary infertility | 372 (60.39%) | 1136 (61.47%) |  |
| Secondary infertility | 244 (39.61%) | 712 (38.53%) |  |
| Basal FSH (IU/L) | 6.61 (5.64, 7.70) | 6.40 (5.48, 7.58) | 0.051 |
| Basal LH (IU/L) | 4.92 (3.58, 6.81) | 5.11 (3.61, 7.20) | 0.177 |
| Basal AFC | 18.00 (13.00, 20.00) | 18.00 (11.00, 22.00) | 0.465 |
| Fertilization type, n (%) |  |  | 0.714 |
| IVF | 406 (65.91%) | 1203 (65.10%) |  |
| ICSI | 210 (34.09%) | 645 (34.90%) |  |
| E2 on trigger Day (pg/ml) | 3409.50 (2294.00, 4902.00) | 2663.00 (1848.00, 4221.00) | <0.001 |
| LH on trigger Day (IU/L) | 2.72 (1.77, 3.93) | 2.28 (1.28, 3.88) | <0.001 |
| P on trigger Day (ng/ml) | 2.17 (0.90, 3.87) | 1.00 (0.62, 1.68) | <0.001 |
| Follicle counts on trigger Day | 15.00 (11.00, 19.00) | 13.00 (9.00, 17.00) | <0.001 |

Data are displayed as mean ± standard deviation and median (interquartile range) for continuous variables and n (%) for categorical variables. *BMI* body mass index, r-*FSH* recombinant follicle-stimulating hormone, *r-LH* recombinant luteinizing hormone, *AFC* antral follicle count, *IVF* in-vitro fertilization, *ICSI* intracytoplasmic sperm injection, *E2* estradiol, *P* progestin.

**Supplementary table 2** Characteristics of the fresh embryo transfer cycles of normal responders

| **Variables** | **r-FSH/r-LH** | **r-FSH** | ***p*** |
| --- | --- | --- | --- |
| No. of cycles | 242 | 726 |  |
| Female age (year) | 29.90 ± 4.29 | 29.97 ± 3.89 | 0.816 |
| Male age (year) | 31.58 ± 5.20 | 31.61 ± 4.46 | 0.944 |
| Female BMI (kg/m^2^) | 22.79 ± 3.34 | 22.93 ± 3.51 | 0.587 |
| Infertility factor, n (%) |  |  | 0.976 |
| Ovulatory disorder | 64 (26.45%) | 200 (27.55%) |  |
| Diminished ovary reserve | 21 (8.68%) | 70 (9.64%) |  |
| Pelvic and tubal disease | 90 (37.19%) | 255 (35.12%) |  |
| Endometriosis | 13 (5.37%) | 39 (5.37%) |  |
| Male factor | 54 (22.31%) | 162 (22.31%) |  |
| Infertility type, n (%) |  |  | 0.849 |
| Primary infertility | 148 (61.16%) | 449 (61.85%) |  |
| Secondary infertility | 94 (38.84%) | 277 (38.15%) |  |
| Basal FSH (IU/L) | 6.69 (5.68, 7.69) | 6.52 (5.61, 7.84) | 0.553 |
| Basal LH (IU/L) | 4.99 (3.48, 6.59) | 4.99 (3.51, 6.97) | 0.607 |
| AFC | 18.00 (12.00, 20.00) | 16.00 (10.00, 23.00) | 0.570 |
| Fertilization type, n (%) |  |  | 0.938 |
| IVF | 157 (64.88%) | 469 (64.60%) |  |
| ICSI | 85 (35.12%) | 257 (35.40%) |  |
| E2 on trigger Day (pg/ml) | 2607.50 (1837.00, 3378.0 | 2204.50 (1574.50, 3180.5 | <0.001 |
| LH on trigger Day (IU/L) | 2.85 (1.90, 4.41) | 2.31 (1.33, 3.89) | <0.001 |
| P on trigger Day (ng/ml) | 1.72 (0.73, 3.16) | 0.79 (0.51, 1.16) | <0.001 |
| Follicle counts on trigger Day | 13.00 (10.00, 15.00) | 12.00 (8.00, 15.00) | <0.001 |

Data are displayed as mean ± standard deviation and median (interquartile range) for continuous variables and n (%) for categorical variables. *BMI* body mass index, r-*FSH* recombinant follicle-stimulating hormone, *r-LH* recombinant luteinizing hormone, *AFC* antral follicle count, *IVF* in-vitro fertilization, *ICSI* intracytoplasmic sperm injection, *E2* estradiol, *P* progestin.

**Supplementary table 3** Characteristics of the FET cycles of normal responders

| **Variables** | **r-FSH/r-LH** | **r-FSH** | ***p*** |
| --- | --- | --- | --- |
| No. of cycles | 510 | 1530 |  |
| Female age (year) | 29.62 ± 3.85 | 29.43 ± 3.62 | 0.318 |
| Male age (year) | 31.56 ± 5.29 | 31.36 ± 4.44 | 0.444 |
| Female BMI (kg/m^2^) | 22.36 ± 2.95 | 22.36 ± 3.33 | 0.988 |
| Infertility factor, n (%) |  |  | 0.928 |
| Ovulatory disorder | 135 (26.47%) | 426 (27.84%) |  |
| Diminished ovary reserve | 25 (4.90%) | 72 (4.71%) |  |
| Pelvic and tubal disease | 214 (41.96%) | 633 (41.37%) |  |
| Endometriosis | 46 (9.02%) | 122 (7.97%) |  |
| Male factor | 90 (17.65%) | 277 (18.10%) |  |
| Infertility type, n (%) |  |  | 0.252 |
| Primary infertility | 316 (61.96%) | 991 (64.77%) |  |
| Secondary infertility | 194 (38.04%) | 539 (35.23%) |  |
| Basal FSH (IU/L) | 6.45 (5.63, 7.58) | 6.37 (5.39, 7.48) | 0.094 |
| Basal LH (IU/L) | 4.82 (3.65, 6.65) | 5.19 (3.60, 7.26) | 0.041 |
| AFC | 18.00 (14.00, 20.00) | 18.00 (12.00, 22.00) | 0.830 |
| Fertilization type, n (%) |  |  | 0.317 |
| IVF | 348 (68.24%) | 1007 (65.82%) |  |
| ICSI | 162 (31.76%) | 523 (34.18%) |  |
| E2 on trigger Day (pg/ml) | 4140.00 (2794.00, 5121.0 | 3291.00 (2254.00, 4956.0 | <0.001 |
| LH on trigger Day (IU/L) | 2.50 (1.66, 3.71) | 2.29 (1.35, 3.81) | 0.007 |
| P on trigger Day (ng/ml) | 2.63 (1.04, 4.20) | 1.17 (0.70, 2.11) | <0.001 |
| Follicle counts on trigger Day | 16.00 (13.00, 20.00) | 14.00 (10.00, 19.00) | <0.001 |

Data are displayed as mean ± standard deviation and median (interquartile range) for continuous variables and n (%) for categorical variables. *FET* frozen-thawed embryo transfer, *BMI* body mass index, r-*FSH* recombinant follicle-stimulating hormone, *r-LH* recombinant luteinizing hormone, *AFC* antral follicle count, *IVF* in-vitro fertilization, *ICSI* intracytoplasmic sperm injection, *E2* estradiol, *P* progestin.

**Supplementary table 4** Characteristics of the complete cycles of normal responders

| **Variables** | **r-FSH/r-LH** | **r-FSH** | ***p*** |
| --- | --- | --- | --- |
| No. of cycles | 470 | 1410 |  |
| Female age (year) | 29.72 ± 4.11 | 29.69 ± 3.74 | 0.869 |
| Male age (year) | 31.49 ± 5.19 | 31.52 ± 4.65 | 0.914 |
| Female BMI (kg/m^2^) | 22.45 ± 3.10 | 22.31 ± 3.33 | 0.415 |
| Infertility factor, n (%) |  |  | 0.937 |
| Ovulatory disorder | 125 (26.60%) | 368 (26.10%) |  |
| Diminished ovary reserve | 33 (7.02%) | 116 (8.23%) |  |
| Pelvic and tubal disease | 192 (40.85%) | 563 (39.93%) |  |
| Endometriosis | 37 (7.87%) | 108 (7.66%) |  |
| Male factor | 83 (17.66%) | 255 (18.09%) |  |
| Infertility type, n (%) |  |  | 0.643 |
| Primary infertility | 281 (59.79%) | 860 (60.99%) |  |
| Secondary infertility | 189 (40.21%) | 550 (39.01%) |  |
| Basal FSH (IU/L) | 6.66 (5.68, 7.80) | 6.49 (5.52, 7.70) | 0.175 |
| Basal LH (IU/L) | 5.00 (3.51, 7.07) | 5.16 (3.70, 7.03) | 0.251 |
| AFC | 18.00 (13.00, 20.00) | 17.00 (11.00, 22.00) | 0.151 |
| Fertilization type, n (%) |  |  | 0.737 |
| IVF | 310 (65.96%) | 918 (65.11%) |  |
| ICSI | 160 (34.04%) | 492 (34.89%) |  |
| E2 on trigger Day (pg/ml) | 3571.00 (2296.50, 4938.0 | 2764.50 (1851.00, 4582.5 | <0.001 |
| LH on trigger Day (IU/L) | 2.61 (1.70, 3.78) | 2.27 (1.36, 3.88) | 0.002 |
| P on trigger Day (ng/ml) | 2.25 (0.91, 3.99) | 1.00 (0.60, 1.81) | <0.001 |
| Follicle counts on trigger Day | 15.00 (11.00, 19.00) | 13.00 (9.00, 18.00) | <0.001 |

Data are displayed as mean ± standard deviation and median (interquartile range) for continuous variables and n (%) for categorical variables. *BMI* body mass index, r-*FSH* recombinant follicle-stimulating hormone, *r-LH* recombinant luteinizing hormone, *AFC* antral follicle count, *IVF* in-vitro fertilization, *ICSI* intracytoplasmic sperm injection, *E2* estradiol, *P* progestin.

**Supplementary table 5** Number of oocytes retrieved and embryo assessment

| **Variables** | **r-FSH/r-LH** | **r-FSH** | ***p*** |
| --- | --- | --- | --- |
| No. of cycles | 616 | 1848 |  |
| Oocyte retrieval | 10.00 (7.00, 12.00) | 10.00 (7.00, 13.00) | 0.005 |
| IVF 2PN number | 7.00 (4.00, 9.00) | 6.00 (4.00, 8.00) | 0.003 |
| IVF 2PN rate (%) | 90.00% (80.00%, 100.00%) | 85.71% (73.33%, 100.00%) | <0.001 |
| ICSI 2PN number | 6.00 (4.00, 9.00) | 6.00 (4.00, 8.00) | 0.518 |
| ICSI 2PN rate (%) | 81.82% (62.50%, 92.86%) | 77.78% (60.00%, 90.00%) | 0.100 |
| Usable embryo | 5.00 (3.00, 7.00) | 5.00 (3.00, 7.00) | 0.307 |
| Usable embryo rate (%) | 91.29% (71.43%, 100.00%) | 87.50% (66.67%, 100.00%) | 0.111 |
| Good-quality embryo | 4.00 (2.00, 6.00) | 4.00 (2.00, 6.00) | 0.752 |
| Good-quality embryo rate (%) | 70.00% (50.00%, 90.00%) | 72.08% (50.00%, 88.89%) | 0.711 |
| Mild/moderate OHSS rate (%) | 3.73% (23/616) | 3.57% (66/1848) | 0.852 |
| Cycle cancellation rate due to OHSS (%) | 25.65% (158/616) | 16.72% (309/1848) | <0.001 |

Data are displayed as median (interquartile range) for continuous variables because they follow the skewed distribution and % (n) for categorical variables. r-*FSH* recombinant follicle-stimulating hormone, *r-LH* recombinant luteinizing hormone, *IVF* in-vitro fertilization, *2PN* 2 pronuclear, *ICIS* intracytoplasmic sperm injection, *OHSS* ovarian hyper-stimulation syndrome.
